# Secretory Vesicles Targeted to Plasma Membrane During Pollen Germination and Tube Growth

**DOI:** 10.3389/fcell.2020.615447

**Published:** 2021-01-21

**Authors:** Huaqiang Ruan, Jiang Li, Ting Wang, Haiyun Ren

**Affiliations:** Key Laboratory of Cell Proliferation and Regulation Biology of Ministry of Education, Center for Biological Science and Technology, Advanced Institute of Natural Science, Beijing Normal University, Zhuhai, China

**Keywords:** secretory vesicles, plasma membrane, exocyst complex, SNAREs, regulation, pollen, F-actin

## Abstract

Pollen germination and pollen tube growth are important biological events in the sexual reproduction of higher plants, during which a large number of vesicle trafficking and membrane fusion events occur. When secretory vesicles are transported via the F-actin network in proximity to the apex of the pollen tube, the secretory vesicles are tethered and fused to the plasma membrane by tethering factors and SNARE proteins, respectively. The coupling and uncoupling between the vesicle membrane and plasma membrane are also regulated by dynamic cytoskeleton, proteins, and signaling molecules, including small G proteins, calcium, and PIP2. In this review, we focus on the current knowledge regarding secretory vesicle delivery, tethering, and fusion during pollen germination and tube growth and summarize the progress in research on how regulators and signaling molecules participate in the above processes.

## Introduction

In seed plants, the production of seeds depends on double fertilization. Mature pollen grains are tri-cellular and composed of two small sperm cells and a large vegetative cell in *Arabidopsis*. One of the two sperm cells fuses with an egg to form a diploid zygote that develops into an embryo, and the other fuses with the polar nucleus to form a primary endosperm nucleus; this process is called double fertilization (Shi and Yang, 2010). The developmental progression of plant double fertilization is well coordinated: it starts with pollen falling on the stigma; the pollen adheres, hydrates, and germinates on the stigma via specific recognition (Sprunck, 2020). Then, the pollen germinates to produce a tubular structure (the pollen tube) that rapidly elongates through polar growth, penetrates the stigma, and grows in style tissues to ultimately deliver the two immotile sperm cells into the ovule to complete double fertilization (Zheng et al., 2018). Since the proper pollen germination and tube growth are essential for two sperm cells transporting to female gametophyte, so exploring the molecular mechanism of pollen germination and pollen tube growth is of great interest in the field.

Pollen germination and pollen tube elongation form the whole process by which polarity is established and maintained. In this process, many cell wall materials, such as pectins and cellulose, are contained as cargo in vesicles with an average diameter of 0.182 μm (Ketelaar et al., 2008; Chebli et al., 2012). These vesicles arise from the Golgi and trans-Golgi network (TGN) and are directionally transported toward and fused with the plasma membrane (PM) at polar exocytosis sites to enable the membrane extension and sustained synthesis of new cell wall material (Wang et al., 2016; Zheng et al., 2018; Grebnev et al., 2020; Guo and Yang, 2020). A better understanding of the molecular mechanisms of pollen germination and tube growth is key for successful sexual reproduction.

The process of pollen germination includes polarity establishment and site determination (Liu et al., 2018), after which the germinated pollen can continue membrane expansion for directional tube growth. Via membrane trafficking and integration, cell wall materials, proteins, and other components for germination and membrane expansion are transported and released; these processes are essential for pollen germination and tube growth (Chebli et al., 2012; Wang et al., 2016). It is generally thought that membrane contact between secretory vesicles and the PM is the most important cellular activity that coordinates pollen germination and pollen tube growth (Gu and Nielsen, 2013; Guo and Yang, 2020). If directional transport- and release-related processes are inhibited, pollen germination and subsequent tube growth and fertilization events will be strongly affected. In recent years, the progress in understanding the dynamic coordination between endocytosis and exocytosis in pollen tubes has been summarized and reviewed (Zhang et al., 2019; Guo and Yang, 2020). In this review, we focus on the process of secretory vesicle directional targeting the PM, which involves vesicle delivery, tethering, and fusion during pollen germination and tip growth, as well as on the different regulators involved, such as some key signaling proteins and other molecules.

## Vesicle Delivery

It is generally thought that vesicles are transported by motor protein-mediated directed transport along microfilaments (MFs) toward the target membrane in plant cells, while in animal cells, the microtubule network serves as the track (Coudrier, 2007; Madison et al., 2015; Ueda et al., 2015; Duan and Tominaga, 2018). Experimental data also suggest that plant MFs maintain greater stability than animal MFs and can withstand long-distance vesicle transport (Ren et al., 2019).

**FIGURE 1:**
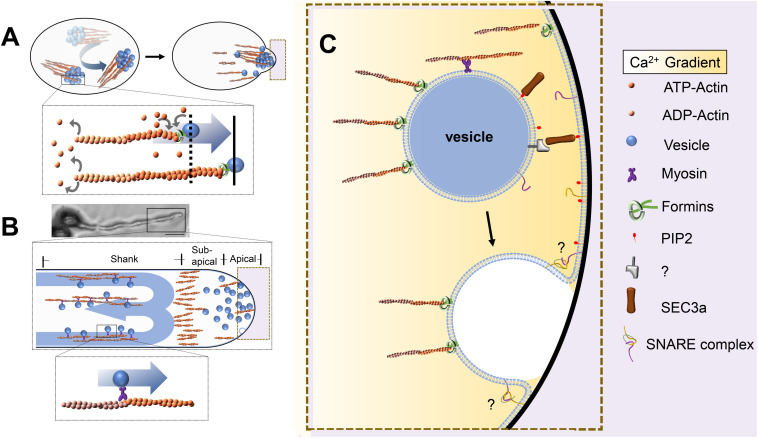
Pattern of vesicle transport in pollen grains and pollen tubes. **(A)** AtFH5 is located to vesicles and promotes actin assembly, the force produced from microfilament polymerization push vesicles’ rotational movement to the potential germination sites. Bottom: The box in the upper is enlarged. **(B)** Vesicles are transported along the microfilaments by moto proteins in the shank region, giving rise to a reverse-fountain cytoplasmic streaming pattern, the microfilaments are arranged into an actin fringe at the subapical region. Top: Pollen grains with pollen tubes. Bar is 20 μm. Middle: The box in the top is enlarged. Bottom: The box in the middle is enlarged. **(C)** An enlarged image of dotted frame in A and B. Vesicles are tethered and fused to the plasma membrane by tethering factors and SNAREs. Objects are not to scale.

Although massive dynamic vesicular transport is dependent on the MF network, the coordination between vesicles and their MF tracks shows quite different dynamic patterns during pollen germination and tube growth (Lan et al., 2018; Liu et al., 2018). Recent research has revealed that actin filaments rotate along the outer edges of pollen grains and then gather in future pollen germination sites, forming collar-like actin structures. Genetic and pharmacological evidence has further revealed an interdependent relationship between the mobility of vesicles and the polymerization of actin filaments. AtFH5, a highly expressed FORMIN protein in *Arabidopsis thaliana* pollen, is located in vesicles and promotes actin assembly; in turn, the force produced by MF polymerization pushes vesicles to the potential germination site (Figure 1A; Liu et al., 2018). In the pollen tube, the actin cytoskeleton shows a well-organized and highly dynamic structure that might correspond to the specific functions in different regions (Figure 1B; Xiang et al., 2007; Fu, 2015). In the shank region, the parallel F-actin cables are thought to serve as tracks for transporting organelles and vesicles to the pollen tube tip (Hepler and Cheung, 2001; Vidali et al., 2001). In the subapical region, the MFs are short and dense and form a collar-like zone, which might be used as a filter to prevent large organelles and other large membrane structures from entering the tip region (Kroeger et al., 2009; Dong et al., 2012; Diao et al., 2020). In the apical region of the pollen tube, actin filaments are highly dynamic and are thought to organize vesicle docking and fusion with the PM of the pollen tube tip (Vidali et al., 2001; Qu et al., 2013). Interestingly, there exist two alternative possible working patterns between F-actin and secretory vesicles during pollen germination and tube growth (Figures 1A,B). However, it is not yet clear how these two coordination patterns work and whether they equally contribute to pollen germination and tube growth. Furthermore, single molecular techniques and *in vitro* simulation assays are expected to be introduced that will help to reveal how single F-actin molecules function on vesicles and elucidate the underlying mechanism.

To ensure that secretory vesicles are delivered to the PM along the correct route, actin filaments need to be temporally and spatially coordinated and arranged in a highly dynamic manner. Different classes of actin-binding proteins (ABPs) are involved in this regulation. Among the ABPs, class I formins are very exciting candidate coordinators of actin and vesicle dynamics, since they can localize to secretory vesicles, bind to F-actin, and directly regulate F-actin dynamics (Deeks et al., 2005; Cvrckova et al., 2014; Li S. et al., 2017; Lan et al., 2018; Liu et al., 2018). Lan’s et al. research showed that MF nucleation factor formins (FORMIN3 and FORMIN5) can localize to the PM at the tip of the pollen tube and initiate MF assembly (Lan et al., 2018). The pollen germination percentage is significantly reduced in *fh3-2 fh5-3* mutant plants. Loss of actin filaments in the pollen tubes of *fh3fh5* mutants reduces the velocity of tip-directed vesicle transport and alters the apical vesicle accumulation pattern, supporting the idea that apical actin filaments and their regulatory formin proteins can regulate vesicle trafficking (Ye et al., 2009; Lan et al., 2018). It would be very interesting to explore deeply whether and how these pollen-expressed formin proteins are involved in vesicle trafficking and integration processes. Another family of ABPs, the profilins, can interact with formins via the FH1–FH2 domain to enhance filament elongation rates and to thin and elongate actin bundles (Zhang et al., 2016; Li S. et al., 2017). Some actin depolymerization factors, such as ADF5 and actin-bundling proteins (i.e., VILLIN2 and VILLIN5), have been reported to affect actin dynamics, further influencing pollen germination and tube growth; however, there is no evidence showing an interaction with vesicle trafficking or vesicle integration (Qu et al., 2013; Zhu et al., 2017; Diao et al., 2020). Although it is known that the dynamic organization of MFs and their regulatory proteins are essential for targeting of vesicles to the PM for secretion, the underlying molecular mechanism is still unclear, especially regarding how actin organization interacts with secretory vesicles and directs vesicle targeting. Which protein families may be involved during this process still needs to be further explored.

Compared to the role of the MF cytoskeleton in vesicle delivery, the roles of microtubules in pollen tubes are less clear. Pollen tubes contain many microtubule motors of the kinesin family, and pollen-expressed kinesin proteins are believed to be involved in the distribution of organelles during pollen tube growth (Cai and Cresti, 2010). Further exploration of the function of the microtubule network, especially the role this network plays in vesicle trafficking during pollen germination and tube growth, would provide more information and help to comprehensively elucidate the role of the cytoskeleton.

Signaling molecules such as the small G protein Rho of plant GTPase (ROP), the second messenger Ca^2+^, and the phospholipid molecule phosphatidylinositol-4,5-bisphosphate (PIP2) play important roles in pollen germination and vesicle transport in pollen tubes (Berken and Wittinghofer, 2008; Steinhorst and Kudla, 2013; Feiguelman et al., 2018). As molecular switches, small G proteins have two forms: an inactivated GDP-bound form and an active GTP-bound form. ROPs are regulated by ROPGEFs, ROPGAPs, and RHOGDIs (Berken, 2006). In *A. thaliana*, ROP1, ROP3, and ROP5 are expressed in pollen tubes. ROPs regulate cytoskeletal dynamics and endocytosis through their downstream effector proteins. The pollen-specific protein ROP1 accumulates in the PM of the top of the pollen tube to regulate the Ca^2+^ concentration gradient, activate the RIC3 pathway, and promote actin depolymerization (Gu et al., 2005; Zhou et al., 2015). Moreover, ROP1 also activates RIC4 to promote actin assembly, change the arrangement of the MF skeleton, and induce the accumulation and transport of vesicles to the tip region (Gu et al., 2005; Lee et al., 2008). These results indicate that ROP1 is involved in vesicle transport through regulation of MFs in pollen tubes. However, it is still unclear whether establishment of pollen polarity is also regulated by ROP during pollen germination.

PIP2 localizes at the pollen tube apical PM. The balance of its distribution and content is very important for maintenance of the normal growth of pollen tubes (Monteiro et al., 2005; Zhang and McCormick, 2010). PI(4,5)P2 is synthesized by PIP5K kinase, and the pollen germination and pollen tube polarity growth of the *pip5k4* homozygous mutant are significantly impaired (Sousa et al., 2008). Ischebeck et al. (2011) found that overexpression of PIP5K10 or PIP5K11 enlarged the tip of the pollen tube and caused abnormal arrangement of the MF cytoskeleton, indicating that PI(4,5)P2 regulates dynamic changes in the MF cytoskeleton. Phosphatidylserine (PS) is abundant in the inverted-cone zone of the apical pollen tube in *Arabidopsis*. Recent research has revealed that loss of apical localization of PS and significantly decreased distribution lead to obvious decreases in vesicle numbers and an obvious increase in pollen tube width, which indicates that tip-localized PS establishment is important for vesicle targeting/trafficking and polar growth of pollen tubes in *Arabidopsis* (Zhou et al., 2020).

Although the oscillation of [Ca^2+^]_cyt_ follows the growth rate pulse, the oscillation of the pollen tube growth rate is consistent with changes in vesicle dynamics (Holdaway-Clarke et al., 1997; Parton et al., 2001). When pollen tubes reach the growth peak, considerable exocytosis is observed at the top of the test tube. Ca^2+^-dependent ABP LILIM1 binds F-actin bundles in lily pollen and protects them from depolymerization under low [Ca^2+^]_cyt_ (Wang et al., 2008). In contrast, with increases in [Ca^2+^]_cyt_, villin/gelsolin family members cut off actin filaments, decrease the activity of profilin, and reduce the polymerization of MFs (Zhang et al., 2010). The above data indicate that Ca^2+^ can indirectly affect vesicle transport in pollen tubes. Whether it can also directly affect cytoskeletal dynamics or intracellular vesicle transport remains to be further studied.

## Vesicle Tethering

After vesicles are delivered in proximity to the target membrane, contact is required between the vesicle and target membrane before fusion, and multisubunit tethering complexes are thought to enable this initial encounter (Figure 1C). The first contact between vesicles and the PM is mediated mainly by the exocyst complex (Yu and Hughson, 2010; Pleskot et al., 2015; Mei et al., 2018). The exocyst complex is composed of the subunits Sec3, Sec5, Sec6, Sec8, Sec10, Sec15, Exo70, and Exo84, which are highly conserved in eukaryotes (TerBush et al., 1996; Mei et al., 2018). There are two models for the mechanism by which the exocyst complex performs its tethering function in yeast and mammalian cells (Yu and Hughson, 2010). In the first model, Sec3 and Exo70 interact directly with P(4,5)P2 on the PM to mark secretion sites (He et al., 2007; Zhang et al., 2008). The remaining six subunits form a subcomplex, which is recruited to the vesicle membrane through interaction between Sec15 and the Rab GTPase Sec4p (Guo et al., 1999). In the other model, all eight subunits of the exocyst complex assemble into two different subcomplexes. One of them is composed of Sec3, Sec8, Sec5, and Sec6, which are anchored to the membrane through Sec3 and directly interact with P(4,5)P2. Another subcomplex consists of Sec10, Sec15, Exo70, and Exo84, which are located on the vesicle. Then, interaction between Sec8 and Sec10 assembles the two subcomplexes into a complete exocyst complex to complete the process of vesicle tethering to the PM (Katoh et al., 2015; Heider et al., 2016; Polgar and Fogelgren, 2018). However, the molecular mechanism of the exocyst complex in plants is still poorly understood. Mutations in plant exocyst subunits, such as *sec6*, *sec15a*, and *sec5a/sec5b* single and double mutations, cause defects in pollen germination and tube growth, while *sec8* and *sec3a* mutations have been reported to cause male-specific transmission defects (Cole et al., 2005; Bloch et al., 2016; Li Y. et al., 2017).

Pollen grain germination has been found to be defective in a *sec3a/SEC3A* heterozygous mutant (Li Y. et al., 2017), while in overexpression lines, multiple tips emerge from pollen grain surfaces, and GFP-SEC3A signals appear only in the PM at the tip of the growing pollen tube (Bloch et al., 2016). These results suggest that SEC3A plays an important role in establishing polarity during pollen germination and tube growth. In addition, in sec3a/GFP-SEC3A-overexpressing complementation lines, a strong positive correlation between the localization of GFP-SEC3A at the tips of growing pollen tubes and the secretion of esterified pectins suggests that GFP-SEC3A might work as an intracellular marker for exocytosis. It would be very interesting to explore how SEC3A and other components coordinate to participate in the vesicle secretion process and which other essential proteins/other molecules are secreted by the exocyst-related pathway. The localization and dynamics of SEC8 in pollen tubes are consistent with those of SEC3A, and the homozygous *sec8*-null mutant also shows defects in male-specific transmission, similar to the *sec3a* mutant (Cole et al., 2005; Hala et al., 2008), which indicates that SEC3A and SEC8 may function together to participate in polar transport and the release of key contents for germination and tube growth. There are 23 potential *EXO70* genes in *Arabidopsis*, and mutations in *EXO70* subunits cause different defects in pollen germination and tube growth (Elias, 2003; Synek et al., 2006, 2017; Li et al., 2010; Vukasinovic and Zarsky, 2016). The diversity of *EXO70* family genes implies that there is a large degree of functional redundancy among the subunits. EXO70C2 seems to play a dominant role together with EXO70C1, since the *exo70c1exo70c2* double mutation causes a complete pollen-specific transmission defect (Synek et al., 2017). The yeast two-hybrid system has identified interactions of *Arabidopsis* SEC3A with EXO70A1, SEC10 with SEC15b, and SEC6 with SEC8; these findings indicate that the plant exocyst complex might be structurally conserved and similar to that in animal and yeast cells (Hala et al., 2008). Given data from dynamic imaging analysis of exocyst components in other plant tissues and cells (e.g., root epidermal cells) and the molecular mechanisms of tethering in other cell types (Fendrych et al., 2013), it is reasonable to hypothesize that the exocyst complex also functions as a tethering complex for vesicle transport during pollen germination and tube growth. In the future, some alternative imaging methods could be developed and applied to pollen cells to track the dynamic pattern of each particle component. The results of such studies would offer us more details about how the exocyst complex functions in tethering secretory vesicles.

In yeast cells, sec3p and Exo70p can be recruited to the PM by binding directly with P(4,5)P2, and other subunits are assembled at the active secretion site by Sec4p (Novick et al., 2006). Similarly, the exocyst complex is also expressed in polar secretory active sites in animal cells (Anitei et al., 2006). Although the interaction of SEC3A and PIP2 in plant cells has been confirmed by *in vitro* assays, *in vivo* analysis of the truncated SEC3A protein with loss of the key PIP2 interaction domain has indicated that the interaction does not affect SEC3A apical PM localization and functionality in *Arabidopsis* (Bloch et al., 2016). However, different results from ectopic expression of the same truncated protein have been obtained in tobacco cells, so the interaction and functionality between PIP2 and exocyst components in plant cells still need to be explored (Bloch et al., 2016; Li Y. et al., 2017).

The Rho small G proteins Cdc42, RHO1, and RHO3 regulate the polar localization of sec3p and exo70p in yeast cells (Finger et al., 1998; Zhang et al., 2008). In plant cells, it has been found that ICR/RIP, an effector protein of ROP, can form a complex with active ROP1 and SEC3A to regulate root cell polar growth (Lavy et al., 2007; Li Y. et al., 2017; Li et al., 2008). It would be very interesting to screen and identify whether some ROP effector or regulatory proteins interact with the exocyst complex and coordinate the tethering process during pollen germination and tube growth.

A general model of exocyst action suggests that most of the components that arrive at the PM and tether secretory vesicles cannot localize properly after disruption of the actin cytoskeleton (Synek et al., 2014). In budding yeast, gene mutations in *SEC10* and *SEC15* strongly affect the cytoskeleton, leading to significant defects in the actin cytoskeleton (Aronov and Gerst, 2004). Inhibition of the interaction between EXO70 and the Arp2/3 complex blocks the formation of actin-based membrane protrusions and affects cell motility in animal cells, which indicates the special role EXO70 might play in coordinating the cytoskeleton and membrane trafficking during cell migration (Zuo et al., 2006). Recent research has revealed that For1F is a fusion protein containing both the exocyst complex subunit (SEC10) domain and the conserved actin-nucleating factor (formin) domain and that this new fusion protein is essential for polar growth in *Physcomitrella patens* (van Gisbergen et al., 2018). This work suggests that both the exocyst complex and actin filaments are essential and cooperate in tethering secretory vesicles during exocytosis. Further exploration of exocyst–cytoskeleton interactions in different cell types would offer some very important clues and elucidate possible cooperative strategies in plant cells, which could help us to better understand the molecular mechanism of vesicle trafficking.

## Vesicle Fusion

**FIGURE 2:**
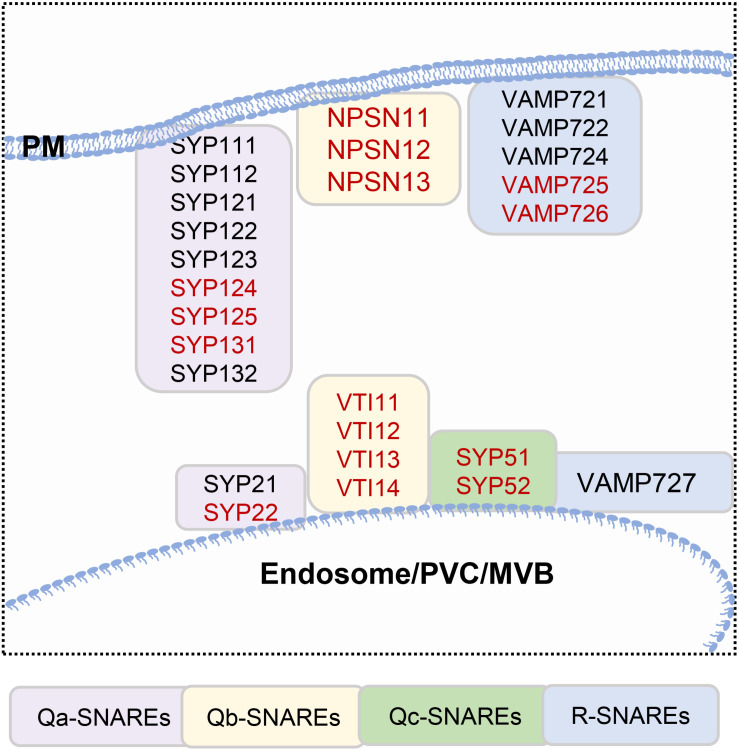
SNAREs located in the plasma membrane and endosomes. Genes that are highly expressed in pollen, which are highlighted in red.

Membrane fusion occurs after the tethering of vesicles and target membranes; soluble *N*-ethylmaleimide-sensitive factor attachment receptors (SNAREs) play a major role in membrane fusion Figure 1C (Uemura et al., 2004; Lipka et al., 2007; Sanderfoot, 2007). SNAREs are classified as Qa-, Qb-, Qc-, and R-SNAREs based on their conserved residues, and all contain a hydrophobic SNARE domain. The SNARE proteins located in the PM and endosomes are listed in Figure 2. The Qa-SNARE family members AtSYP124, AtSYP125, and AtSYP131 are exclusively expressed in male gametophytes (Silva et al., 2010; Ichikawa et al., 2015; Slane et al., 2017). The *syp124syp125syp131* mutant shows more severe male gametophyte defects than the *syp124syp125* double mutant, and the pollen tube stops growing during passage through the style, suggesting functional redundancy (Silva et al., 2010; Ichikawa et al., 2015; Slane et al., 2017). SYP131 is mainly stably located in the PM, while SYP124/SYP125 seems to circulate between the PM and endosomes. Therefore, SYP124 and SYP125 may be responsible for membrane fusion in the recycling pathway, while SYP131 may preferentially mediate the membrane fusion of secretory vesicles and contribute to the growth of pollen tubes (Silva et al., 2010; Ichikawa et al., 2015; Slane et al., 2017). VAMP72 family proteins are plant-specific R-SNARE proteins that are located mainly in the PM (Lipka et al., 2007). It has been found that the pollen tubes of *vamp721*^+ /−^ *vamp722*^+ /−^ show a certain proportion of curly phenotypes, and half of them lack the Ca^2+^ channel AtCNGC18 on the PM (Meng et al., 2020). The N-terminal longin domains of AtVAMP721 and AtVAMP722 interact with AtMLO5, recruit AtCNGC18 to relocate to the PM, affect the local cytoplasmic Ca^2+^ concentration, and regulate the directional responses of pollen tubes to extracellular signals (Meng et al., 2020). However, research on SNAREs has been scarce; thus far, there have been no reports about Qb- and Qc-SNARE proteins in pollen. The specific members of the SNARE complex that are expressed in pollen and their functions in pollen germination and tube growth are unclear.

Many studies have shown that Ca^2+^ plays an important regulatory role in vesicle fusion (Konopka-Postupolska and Clark, 2017). In *Arabidopsis*, the localization of Qa-SNARE in pollen is also regulated by Ca^2+^ ions. The polarity establishment of SYP125 before germination seems to be related to the establishment of a Ca^2+^ gradient, and the location of SYP124 and SYP125 is also changed while Ca^2+^ flux is disturbed, suggesting that Ca^2+^ regulates vesicle fusion in many ways (Silva et al., 2010; Ichikawa et al., 2015). In addition, the distribution of SYP124 and SYP125 is closely related to MFs, MFs depolymerization destroys their localization (Silva et al., 2010; Ichikawa et al., 2015); and some actin or actin-related proteins have been identified in a interactome analysis of SNARE proteins (Fujiwara et al., 2014). In animal cells, synaptotagmin (SYT) proteins have been reported to regulate vesicle fusion (Kweon et al., 2019). There are seven SYTs in *Arabidopsis* (Ishikawa et al., 2020). In the plant SYT family, SYT1 is the most extensively characterized protein; it acts as an endoplasmic reticulum (ER)–PM tethering factor and participates in biotic and abiotic stress responses in plants (Schapire et al., 2008; Siao et al., 2016). SYTs contain conserved C-terminal tandem C2A and C2B domains and interact with phosphatidylinositol, SNAREs, and Ca^2+^ channel proteins to regulate endocytosis/exocytosis (Wu et al., 2014). It will be interesting to study whether plant cells implement a similar regulatory mechanism in the processes of pollen germination and tube growth. SYT2 is expressed mainly in *Arabidopsis* pollen and is located in the Golgi and PM, and the SYT2-C2AB domain binds to the phospholipid membrane in a Ca^2+^-dependent manner (Wang et al., 2015). The pollen germination rate of *syt2* mutants is decreased, and pollen tube elongation is restricted (Wang et al., 2015), but the relationship between SYT2 and vesicle fusion during pollen germination and pollen tube growth needs to be further confirmed. Annexin, a Ca^2+^ channel protein, can bind to membrane phospholipids in a Ca^2+^-dependent manner and can also bind MFs. Thus, it may provide an important connection among intracellular Ca^2+^ signaling, the actin cytoskeleton, and the membrane and participate in intracellular vesicle trafficking (Konopka-Postupolska and Clark, 2017). Ann5 is a Ca^2+^ channel protein that is highly expressed in mature pollen grains and pollen tubes of *A. thaliana*, and a decrease in its expression leads to severe sterility (Lichocka et al., 2018). Ann5 seems to participate in pollen development, germination, and pollen tube elongation by promoting Ca^2+^-regulated intimal transport, but the exact mechanism needs to be further studied (Zhu et al., 2014a,b; Lichocka et al., 2018).

## Prospects

Pollen germination and pollen tube growth are important biological processes in plant sexual reproduction. Many vesicle trafficking, tethering, and fusion events take place during polar pollen germination and tube elongation. In past years, some key tethered factors and SNARE family members have been identified and characterized. However, there are still some key issues that have not yet been resolved, such as the molecular mechanism of each subunit of the exocyst complex and each member of the SNARE family during pollen germination and pollen tube growth. Recent studies have revealed that actin filaments not only participate in intracellular vesicle transport as tracks but also provide the driving forces for vesicle trafficking. Further study is needed to determine whether and how MFs function in vesicle tethering and fusion with the PM and to reveal the interplay between these processes. In addition, Ca^2+^ is an important signaling molecule for pollen germination and tube growth (Iwano et al., 2004; Hepler et al., 2012; Steinhorst and Kudla, 2013); thus, it will be very meaningful to study which and how Ca^2+^ channels or calcium binding proteins are involved in the regulation of vesicle fusion during pollen germination and pollen tube growth. It is believed that the development of microscopic technologies and research methods will enable in-depth analysis of vesicle delivery, tethering, and fusion to the PM during pollen germination and pollen tube growth.

## Author Contributions

HR drew the figure and wrote the third part “vesicle fusion.” JL wrote the first and second part of the manuscripts “vesicle delivery” and “vesicle tethering.” TW wrote and revised the manuscript. HR designed and organized the concept of the manuscript and revised the manuscript. All authors contributed to the article and approved the submitted version.

## Conflict of Interest

The authors declare that the research was conducted in the absence of any commercial or financial relationships that could be construed as a potential conflict of interest.
